# Female and Rural School Students Show More Positive Attitudes toward Disability during Physical Education Lessons

**DOI:** 10.3390/ijerph19105881

**Published:** 2022-05-12

**Authors:** Jorge Rojo-Ramos, Alejandro Vega-Muñoz, Nicolás Contreras-Barraza, Sabina Barrios-Fernandez

**Affiliations:** 1 Social Impact and Innovation in Health (InHEALTH) Research Group, Faculty of Sport Sciences, University of Extremadura, 10003 Cáceres, Spain; jorgerr@unex.es (J.R.-R.); sabinabarrios@unex.es (S.B.-F.); 2 Public Policy Observatory, Universidad Autónoma de Chile, Santiago 7500912, Chile; 3 Facultad de Economía y Negocios, Universidad Andres Bello, Viña del Mar 2531015, Chile

**Keywords:** attitudes, inclusion, disabilities, physical education

## Abstract

Physical education (PE) lessons offer an excellent opportunity to encourage participation and learning for students with and without disabilities. However, there are still barriers that prevent educative inclusion (EI) from being achieved, with negative attitudes being one of the major issues. This study aimed to explore students without disabilities’ attitudes toward their peers with disabilities in the second stage of Primary Education, examining possible differences according to sex (male or female) and school location (urban or rural). The Scale of Attitudes toward Students with Disabilities in Physical Education–Primary Education (SASDPE-PE), a four-item instrument with a five-point Likert scale ranging from 1 (strongly disagree) to 5 (strongly agree), was administered to 545 Spanish students aged 9 to 12 years old. The results revealed that girls and students from rural schools showed more positive attitudes toward their peers with disabilities during the PE lessons; differences were significant in both cases. Thus, according to our findings, the SASDPE-PE is a practical tool to assess attitudes, even after an attitude-change programme. Furthermore, PE attitude-change programmes should be implemented, especially considering male students and those enrolled in urban schools.

## 1. Introduction

The study of attitudes toward disability has important implications, as it is essential for the success of initiatives and policies. Hence, society’s level of knowledge, beliefs, and attitudes towards disability, defined as negative, stigmatising, or unrealistic attitudes, represents a major barrier to the social inclusion of people with disabilities. Thus, appropriate actions must be undertaken to build effective inclusion [1,2,3,4]. The study of attitudes towards inclusive education (IE) must consider perspectives from all educational stakeholders, including future teachers [5,6,7,8], teaching staff [9,10,11,12,13], children with disabilities’ peers [14,15,16,17], and families [18,19,20]. 

The analysis of attitudes is essential for transforming schools into meeting places for people with disabilities and other kinds of diversity: social, ethnic, cultural, sexual, and linguistic [21,22,23]. Attitude studies are not a new topic, as different authors have proposed their models, definitions, assessment tools, and attitude-change programmes [24,25,26,27,28]. Attitudes can be considered mediators between environmental input and behavioural responses to a reference object, triggering an individual’s response [2,29]. Attitudes cannot be observed directly, so they are inferred from people’s verbal or behavioural manifestations [1]. This study will contextualise attitudes under Triandis’ three-dimensional model [29], which understands attitudes as a predisposition to behave in a certain way in social situations, being composed of three elements: (1) cognitive, related to thoughts, ideas, beliefs, opinions, or perceptions about an attitudinal object; (2) affective, referring to the positive or negative emotional charge when exposed to the attitudinal object; and (3) behavioural, concerning the readiness to act in a certain way. Moreover, it is necessary to consider whether attitudes are explicit or implicit [30,31]: the first refers to the relatively controlled, deliberate, and conscious judgements when interacting with the attitudinal object, while the second is automatic, quicker, difficult to control, and usually involuntarily formed [32,33,34].

The International Charter of Physical Education, Physical Activity and Sport, in their original version (1978) and further revisions (1991, 2015), calls, from a rights-based perspective, for inclusive access to physical education (PE), physical activity (PA), and sports for all citizens, and sets ethical and quality standards for the actors involved in the design, implementation, and evaluation of sports programmes and policies [35]. Moreover, Quality PE (QPE) should ensure all students participate during PE lessons and PA as an extracurricular or leisure activity [36]. Furthermore, QPE has the potential to create planned, progressive, and inclusive learning opportunities for the development of the physical, cognitive, social, and emotional skills required for a physically active life [37].

The last World Health Organization (WHO) guidelines concerning PA and sedentary behaviour stated that school-age children should perform at least an average of 60 min per day of moderate- to vigorous-intensity activity, mostly aerobic, across the week; vigorous-intensity aerobic activities and those that strengthen muscle and bone should be carried out at least three days a week [38]. A QPE is an opportunity to improve the PA level of students with disabilities, as in most cases, they do not comply with these recommendations, presenting sedentary lifestyles leading to health risks [39,40].

Students with disabilities have lower social participation than their typically developing peers; they have fewer friends and are less accepted, have fewer interactions with peers, and usually report feelings of loneliness [14,41,42]. Thus, positive peer attitudes toward students with disabilities are needed for IE. One of the barriers that students with disabilities experience is their peers’ negative attitudes. Sometimes, peers feel uncomfortable, avoid eye contact, or use avoidant body language [43]; these attitudes can lead to low satisfaction when interacting with students with disabilities, preventing future exchanges [15]. Considering the relevance of analysing attitudes and promoting QPE lessons to encourage participation and learning for all students, this study aims to explore the students with typical development attitudes toward their peers with disabilities, to check whether there are differences between the sexes or between students attending urban or rural centres.

## 2. Materials and Methods

### 2.1. Study Design and Ethical Considerations

A cross-sectional study design was used. The study was conducted according to the guidelines of the Declaration of Helsinki and approved by the Ethics Committee of the EDUCA platform for excellence in education research (approval code: 42022).

### 2.2. Participants

The sample consisted of 545 students attending the second stage of Primary Education at public schools in the Autonomous Community of Extremadura (Spain). The eligibility criteria included participants aged 9 to 12 years who provided signed informed consent to the research team.

The participants’ mean age was 11.03 years (SD = 0.81). The variables of sex (male or female), school location (rural or urban), and grade (fifth or sixth grade in Primary Education) were reasonably balanced, as shown in Table 1. 

The threshold to determine whether localities were rural or urban was set at 20,000 inhabitants, as considered by the Cáceres Provincial Council’s website (https://www.dip-caceres.es/, accessed on 24 February 2022). The participants were selected using convenience sampling, a non-probability sampling method [44].

### 2.3. Procedure 

The sample was obtained through the Regional Ministry of Education and Employment public schools database from the Regional Government of Extremadura. All of the centres teaching Primary Education were selected. An email was sent to the school headteachers explaining the aim of the study and asking for their collaboration. Finally, 9 urban and 11 rural schools across Extremadura agreed to participate. The centres were provided with informed consent forms to be signed by parents or legal guardians. Once the centres had compiled the consent forms, they contacted the research team to arrange an in-person appointment to administer the surveys to the students. The questionnaires were developed using Google Forms and were administered via tablet during PE lessons. To ensure understanding of the items, the researchers read each item and resolved students’ doubts. All data were recorded anonymously.

### 2.4. Instruments

The Scale of Attitudes toward Students with Disabilities in Physical Education–Primary Education (SASDPE-PE) was used to assess Spanish students with typical development attitudes toward disability during PE lessons [45]; its Spanish version is available in Appendix A. This instrument comprises four items with the introductory phrase: “During PE lessons, concerning people with disabilities…”. This test covers the behavioural component of the attitudes (Table 2). 

The SASDPE-PE was validated for children aged 9–13 years old from the original SASDPE [46], developed for students between 14 and 19 years old. The responses are collected using a five-point Likert scale ranging from 1 (strongly disagree) to 5 (strongly agree). Thus, higher scores show worse attitudes toward disabilities. The original authors reported a one-factor structure after a confirmatory factor analysis with excellent goodness-of-fit indices and an acceptable reliability value using Cronbach’s alpha <0.79. A correlational study revealed moderate and positive correlations between all items without overlapping [47]. 

### 2.5. Statistical Analysis

All items were reversed before the analyses, so higher scores reveal more positive attitudes toward their peers with disabilities. The Statistical Package for the Social Sciences version 25 for Mac (IBM SPSS, Chicago, IL, USA) was used. As continuous variables did not follow a normal distribution after carrying out the Kolmogorov–Smirnov test, non-parametric statistics were used. The data for categorical variables were presented as mean and percentage, and continuous variables as median and interquartile range. The Mann–Whitney U test was used to analyse the differences between the SASDPE-PE items and their total score according to students’ sex and school location. Thus, to establish the significance level, the Bonferroni correlation was applied for the p-value and set at *p* < 0.016. Cronbach’s alpha was used to estimate the instrument’s reliability, considering values between 0.70 and 0.90 satisfactory [47].

## 3. Results

Table 3 shows the SASDPE-PE scores according to students’ sex and school location using the Mann–Whitney U test. Significant differences were found in items 2 and 3, with higher values in female students and those in rural schools.

After performing a descriptive analysis and searching for differences according to students’ sex and school location, the results showed that female students scored higher than males, and students in rural schools scored higher than those in urban schools, revealing more positive attitudes toward disability. In both cases, the differences were significant (Table 4).

Finally, the SASDPE-PE reliability using Cronbach’s alpha was 0.83, which is considered a satisfactory value [47].

## 4. Discussion

### 4.1. Main Findings and Theoretical Implications 

This research contributes to the knowledge about attitudes toward disability. The Scale of Attitudes toward Students with Disabilities in Physical Education–Primary Education (SASDPE-PE), a four-item instrument with a five-point Likert scale from 1 (strongly disagree) to 5 (strongly agree), was administered to 545 Spanish students aged 9 to 12 years old. Female pupils and students attending rural schools (less than 20,000 inhabitants) showed more positive attitudes than males and students in urban schools. 

The SASDPE-PE [45] is the adapted version for Primary Education from the Scale of Attitudes toward Students with Disabilities in Physical Education (SASDPE) [46], developed for students between 14 and 19. The SASDPE was validated with 609 Spanish students. A confirmatory factor analysis confirmed a unidimensional solution with excellent fit indices. Finally, it was composed of four items about the behavioural component of attitudes and was found to be invariant with sociodemographic variables. The original authors reported acceptable reliability (≥0.77) using Cronbach’s alpha [47]. The SASDPE-PE was validated with 465 Spanish students aged 9 to 13 years. The authors confirmed adequate goodness-of-fit indices, with the factor structure kept invariant concerning grade, sex, and previous contact with individuals with disabilities. Reliability was 0.79, an acceptable value [47]. Several studies have used these tools to study attitudes toward disability during PE lessons to identify attitudes’ antecedents and consequences, and as a tool to assess attitude-change programmes or inclusive methodologies [46]. An exploratory study was carried out using the SASDPE with Spanish students aged 14–16 years who had contact with individuals with intellectual disability (ID) to assess whether inclusive PE (*n* = 25) or a visit to the ID users’ work facilities (*n* = 25) improved students’ positive attitudes toward disability. A control group (*n* = 33) was also included, showing significant differences between groups. In another experiment involving 51 students aged 10–11 years, the SASDPE was used to test whether students’ attitudes toward disability in PE classes improved by playing different cooperative-sensitising games. There were no significant differences in the test–retest total score, but significant differences were found in items 1 and 3, suggesting more positive attitudes in those aspects [48]. Another study using the boccia was carried out with 28 students aged between 12 and 16 years, revealing a slight worsening in attitudes according to the SASDPE results, although not significative, while the rest of the measures improved [49]. Another study with the SASDPE was performed with students between 12 and 18 years old to explore differences in sex, previous contact, and skills and competence perception; significant differences showed that female students, younger students, and those with contact with a classmate with a disability had more positive attitudes [50].

Regarding sex, our study shows significant differences, suggesting more positive attitudes in female students. This finding is in line with the abovementioned study [50]. Research using other assessment tools found similar outcomes. Another study explored the association between having had contact with special educational needs (SEN) students. Students with SEN were less frequently nominated by their peers for joint activities. Female students were nominated more often than males, and, in general, girls showed better attitudes toward disability [14]. Another work using the Multidimensional Attitudes Toward People with Disabilities Scale was administered to 1525 Polish students aged 12–15 years old and revealed that girls have significantly positive attitudes toward students with disabilities in the case of cognitive and affective attitude elements. However, in the case of the behavioural element, there were no differences between the sexes [15]. Furthermore, a literature review explored sex differences in attitudes toward peers with disabilities, with girls presenting more positive attitudes than boys [16].

Studies comparing attitudes between urban and rural students are scarcer. Most work has focused on exploring university students’ [51,52,53] and teachers’ [54,55,56,57] attitudes. One study conducted with 339 primary, secondary, and university students explored the solidarity and acceptance attitudes of students with disabilities; they considered urban and rural locations but found no significant differences [58].

PE lessons are an opportunity to promote students with disabilities’ health and active participation. QPE helps them to comply with the WHO recommendations and strengthen their social status by participating in community activities [37]. Physical inactivity is a serious global health problem, and its associations with obesity or obesity-related diseases are well documented: children with disabilities are less physically active, tend to adopt a more sedentary lifestyle, and are at three to six times greater risk of obesity [40,59,60]. Moreover, student participation is aligned with goals 3, “Health and well-being”; 4, “Quality education”; and 10 “Reducing inequalities” within the 2030 Agenda [61,62]. Furthermore, countries that have ratified the Convention on the Rights of the Child [63] and the Rights of Persons with Disabilities [64], such as Spain, are called upon to encourage all students’ participation during PE lessons and in all school activities and community events.

### 4.2. Practical Implications

According to our data and other studies’ results, female students show more positive attitudes toward their peers with disabilities than males, and students in rural centres have more positive attitudes than those in urban schools. These findings reinforce that attitude-change programmes should be implemented in schools, focusing on males and students in urban locations. PE attitude-change programmes often include inclusive PA, adapted sports, cooperative games, and didactic units with students with and without disabilities participating together. 

Moreover, the SASDPE-PE is a quick and easy-to-use tool that characterises the behavioural element during PE lessons and reports variations after an attitude-change programme. Students with disabilities have the right to participate and learn during QPE lessons, have quality interactions, and enjoy different and inclusive leisure activities like the rest.

### 4.3. Limitations 

Some of the limitations of this study are the following. On the one hand, the results should be interpreted with caution, as the sample was one of convenience. On the other hand, online questionnaires were used for data collection. Although online questionnaires have advantages, such as reducing costs or facilitating data collection and processing, they have disadvantages, such as the probability of sampling bias, failing to know the characteristics of non-respondents, and having a lower response rate [65,66,67,68]. 

## 5. Conclusions

Female students and those students attending rural schools showed more positive attitudes toward their peers with disabilities during PE lessons. Significant differences were found in both sex and school location. Thus, PE attitude-change programmes should be implemented, especially considering male students and those attending urban schools.

Moreover, the SASDPE-PE resulted in a practical and easy-to-use tool to assess students’ attitudes.

## Figures and Tables

**Table 1:** Sociodemographic characteristics of the sample (*n* = 545).

Variable	Categories	*n*	%
Sex	Boys	277	50.8
	Girls	268	49.2
School location	Rural	303	55.6
	Urban	242	44.4
Grade	Fifth	282	51.7
	Sixth	263	48.3

*n*: number; %: percentage.

**Table 2:** The Scale of Attitudes toward Students with Disabilities in Physical Education–Primary Education (SASDPE-PE).

In physical education, concerning people with disabilities:
Item 1. I prefer not to interact with people with disabilities. Item 2. I would avoid doing classwork with a person with a disability. Item 3. I would prevent a person with a disability from joining my team. Item 4. I would not propose a person with a disability as captain of my team.

**Table 3:** Scores for the Scale of Attitudes toward Students with Disabilities in Physical Education–Primary Education (SASDPE-PE) items according to sex and school location.

SASDPE-PE Items	Sex			School Location		
	Girls Boys			Rural Urban		
	M_e_ (IQR)	M_e_ (IQR)	*p*	M_e_ (IQR)	M_e_ (IQR)	*p*
I prefer not to interact with people with a disability	5 (1)	5 (1)	0.830	5 (0)	5 (1)	0.951
I’d avoid doing classwork with a peer with a disability	5 (1)	5 (1)	<0.001 *	5 (1)	4 (2)	<0.001 *
I would prevent a peer with a disability from joining my team	5 (1)	5 (2)	<0.001 *	5 (1)	4 (1)	0.004 *
I would not propose a peer with a disability as captain of my team	5 (1)	4 (2)	0.021	5 (1)	4 (2)	0.197

M_e_: median; IQR: interquartile range; *: Mann–Whitney U test was significant <0.016. SASDPE-PE scores were based on a Likert scale where 1: “strongly agree”; 2: “agree”; 3: “indifferent”; 4: “disagree”; and 5: “strongly disagree”.

**Table 4:** Descriptive analysis and differences for the Scale of Attitudes toward Students with Disabilities in Physical Education–Primary Education (SASDPE-PE) total score.

	Total	Sex			School Location		
		Girls	Boys	*p*	Rural	Urban	*p*
	M_e_ (IQR)	M_e_ (IQR)		M_e_ (IQR)			
SASDPE-PE total scores	4.75 (1)	4.75 (0.75)	4.5 (1.25)	0.001 *	4.75 (0.75)	4.25 (1.25)	<0.001 *

M_e_: median; IQR: interquartile range; *: Mann–Whitney U test was significant <0.016. SASDPE-PE scores were based on a Likert scale where 1: “strongly agree”; 2: “agree”; 3: “indifferent”; 4: “disagree”; and 5: “strongly disagree”.

## Data Availability

The datasets used during the current study are available from the corresponding author upon reasonable request.

## Appendix A

**Table A1:** The Attitudes′ Scale toward Students with Disabilities in Physical Education—Primary Education (SASDPE-PE) Spanish original version: Escala de Actitud hacia el Alumnado con Discapacidad en Educación Física para la Etapa de Educación Primaria (EAADEF-EP).

En educación física, con respecto a las personas con discapacidad…
Ítem 1. Prefiero no relacionarme con personas con discapacidad. Ítem 2. Evitaría hacer un trabajo de clase con una persona con discapacidad. Ítem 3. Evitaría para mi equipo a una persona con discapacidad. Ítem 4. No propondría como capitán de mi equipo a una persona con discapacidad.
